# Promoting GSDME expression through DNA demethylation to increase chemosensitivity of breast cancer MCF-7 / Taxol cells

**DOI:** 10.1371/journal.pone.0282244

**Published:** 2023-03-03

**Authors:** Weihua Gong, Panpan Fang, Maodong Leng, Ying Shi

**Affiliations:** 1 Zhengzhou Key Laboratory of Children’s Infection and Immunity, Children’s Hospital Affiliated to Zhengzhou University, Zhengzhou, China; 2 Clinical Laboratory, The Third Affiliated Hospital of Zhengzhou University, Zhengzhou, China; Western Sydney University, AUSTRALIA

## Abstract

**Objective:**

Breast cancer is the most common and high-incidence cancer in women. It is mainly treated by surgery combined with chemoradiation. The main challenge in treating breast cancer patients is developing resistance to chemotherapeutics, so it is urgent to find potential strategies that can improve the chemotherapy effect of patients. In this study, we aimed to explore the role of GSDME methylation in the sensitivity of chemotherapy for breast cancer.

**Methods:**

Here, we identified breast cancer MCF-7 / Taxol cells models using quantitative real-time PCR (qRT-PCR), Western blotting (WB), and cell counting kit-8 (CCK-8) analyses. Epigenetic changes in it were detected by Methylated DNA immunoprecipitation-sequencing and methylation-specific PCR. The expression level of GSDME in breast cancer cells was observed by qPCR and WB analyses. CCK-8 and colony formation assay were used to detect cell proliferation. Finally, pyroptosis was detected by LDH assay, flow cytometry, and WB analyses.

**Results:**

Our results indicate that ABCB1 mRNA and p-GP expression are significantly increased in breast cancer MCF-7 / Taxol cells. GSDME enhancer methylation was found in drug-resistant cells and was associated with the down-regulation of GSDME expression. After treatment with decitabine (5-Aza-2’-deoxycytidine), the demethylation of GSDME induced the occurrence of pyroptosis and thereby inhibited the proliferation of MCF-7 / Taxol cells. We found that the upregulation of GSDME enhances the chemosensitivity of MCF-7 / Taxol cells to paclitaxel by inducing pyroptosis.

**Conclusion:**

Taken together, we identified decitabine increases GSDME expression through DNA demethylation and induces pyroptosis, thus increasing the chemosensitivity of MCF-7 / Taxol cells to Taxol. Use of decitabine / GSDME / pyroptosis-based treatment strategies may be a new way to overcome the resistance of breast cancer to paclitaxel chemotherapy.

## Introduction

Breast cancer is the most common cancer among women worldwide [1] and is the main cause of cancer-related death [2], with its incidence increasing year by year. Clinically, breast cancer is mainly treated by surgery, radiotherapy, chemotherapy and targeted therapy [3, 4]. However, most patients are still treated with conventional surgery combined with chemoradiation. Among them, chemotherapy is deemed to be the key link to avoiding postoperative cancer recurrence. Taxol is a broad-spectrum antitumor drug and one of the most widely used chemotherapeutics for breast cancer [5, 6]. It interferes with the disintegration of microtubules in tumor cells, causing the cell cycle to stagnate, preventing cancer cells from replicating, and ultimately leading to cell death [7]. Unfortunately, with the widespread use of paclitaxel, breast cancer patients have developed resistance to it, resulting in treatment failure [8–10]. Therefore, it is of urgent importance to seek a potential mechanism for the development of drug resistance and enhance the sensitivity of breast cancer patients to Taxol.

Gasdermin (GSDMs) is a family of pore-forming proteins that play an important part in cell death. GSDME is a fellow of the Gasdermin family. It was originally identified as DFNA5 (deafness, autosomal dominant 5) [11, 12] and was also named ICERE-1 because of its low expression in the estrogen receptor [1]. Recently, more and more studies have shown that GSDME plays a major role in regulating cell death [2, 13, 14]. With the in-depth study of the relationship between the Gasdermin family and anticancer properties, more and more researchers believe that GSDME is an important predictive marker for a variety of cancers [13, 15, 16]. As a tumor suppressor, GSDME has been demonstrated to inhibit cancer cell proliferation, migration, and differentiation [2, 13, 15]. As the research deepens, we have discovered that the lack of GSDME expression may be related to tumor chemotherapy resistance [16–18]. Moreover, loss of GSDME expression has been proposed to cause resistance to etoposide in certain melanoma cells [18]. However, no studies have investigated the relationship between GSDME expression and chemotherapy resistance in breast cancer. We aim to test the feasibility of regulating the expression of GSDME may be an effective method to improve the breast cancer chemotherapy effect and reduce drug resistance.

Abnormal gene expression is a characteristic of human cancer, and changes in DNA methylation status may have profound effects on gene expression. DNA methylation is one of the most stable epigenetic modifications in mammals [19]. It occurs mainly at the CpG dinucleotides where the genome is sparsely distributed [20]. Studies have shown that aberrant DNA methylation is not only associated with human diseases but also a promising biological candidate marker [21]. In this study, we performed immunoprecipitation by enriching methylated DNA fragments using 5-methylcytosine antibody. This method can rapidly identify CpG sites. This, combined with high-throughput sequencing, is considered to be a genome-wide technique for quantifying methylation levels. Many studies have shown that DNA methylation reduces the expression of GSDME in most tumor cells, making it difficult to induce pyroptosis in tumor cells [15, 17, 22, 23]. Therefore, we can use the DNA methyltransferase inhibitor—decitabine to increase the expression of GSDME in certain cancer cells (such as gastric cancer, colorectal cancer, breast cancer, etc.) to increase the sensitivity of its to chemotherapy drugs [24, 25].

Chemotherapy can cause tumor cells death in a variety of ways, one of which is pyroptosis. Pyroptosis, also known as inflammatory cell necrosis, is a newly discovered programmed cell death accompanied by inflammation [26]. Recently, Shao Feng’s group redefined it as procedural necrosis mediated by the gasdermin family [27]. Pyroptosis is closely connected with various human diseases, especially cancer. As research progresses, the role of pyroptosis in tumors has become increasingly prominent. As is known to all, caspase-3, as a classic protein for apoptosis, has been extensively studied. However, recent studies have shown that chemotherapeutic drugs induce pyroptosis of tumor cells [28], in which activated caspase-3 cleave specific sites of pyroptosis-related protein GSDME to generate a GSDME-N terminal domain (S1 Fig). The N-segment domain has lipid-affinity capable of forming pores in the cell membrane, causing cells swelling and releasing cellular contents such as lactate dehydrogenase (LDH) and proinflammatory cytokines [29, 30]. That is to say, chemotherapeutic agents can induce caspase-3-mediated pyroptosis in GSDME-positive cell lines. Targeting GSDME-mediated pyroptosis may improve the chemotherapy sensitivity of cancer cells. In summary, the purpose of this paper is to increase the sensitivity of breast cancer-resistant cells to Taxol and explore the mechanism of its resistance, providing a new direction for the clinical treatment of breast cancer.

## Materials and methods

### Cell culture

The human breast cancer cells (MCF-7) were purchased from the American Type Culture Collection (ATCC, Manassas, USA). Using a low concentration gradient induction method to establish the Taxol-resistant MCF-7 /Taxol cells. MCF-7 cells were cultured in a complete RPMI 1640 (Solarbio, China) medium containing 10% fetal bovine serum (BI, USA), 100 U• ml^-1^ penicillin and 100 mg • ml^-1^ streptomycin. MCF-7 /Taxol cells were cultured stably in complete RPMI 1640 culture medium containing 85.50 nmol• L^-1^ Taxols (Xi ’an Haoxuan bio-tech Co., Ltd., China). The cell lines were cultured in a drug-free medium for more than two weeks before in vitro determination. All cells were aseptically cultured in a humidified atmosphere of 5%CO_2_ at 37℃. The processing time and agent concentration are shown in the figures and/or the corresponding legends.

### Construction of Taxol-resistant cell lines for breast cancer

We used a low concentration gradient induction method to establish the Taxol-resistant MCF-7 /Taxol cells [31]. MCF-7 cells during logarithmic growth were seeded in a 25cm^2^ six-well culture plates and cultured for 12h. Taxol was added following cell adherence. MCF-7 cells were induced with 0.2 times IC50, or 1.60 nmol• L^-1^ Taxol, as the starting concentration. When cells grow steadily at this concentration, Taxol concentration is gradually increased (as follows:1.60 nmol• L^-1^, 2.40 nmol• L^-1^, 3.60 nmol• L^-1^, 5.40 nmol• L^-1^, 8.10 nmol• L^-1^, 12.15 nmol• L^-1^, 18.23 nmol• L^-1^, 27.34 nmol• L^-1^, 38.00 nmol• L^-1^, 57.00 nmol• L^-1^, 85.50 nmol• L^-1^). Finally, MCF-7 /Taxol cells grew steadily in the complete culture medium of 85.50 nmol• L^-1^ Taxol.

### Cell proliferation assay

The proliferation ability of tumor cells was detected by CCK-8 assay according to the manufacturer’s instructions. Briefly, MCF-7 cells and MCF-7 / Taxol cells in the logarithmic growth phase were seeded onto 96-well cell culture plates at a density of 8×10^3^ cells per well in 100 mL RPMI 1640 medium supplemented with 10% fetal bovine serum. When the confluence of cells reaches 70–80%, the culture medium was replaced with fresh medium containing a certain concentration of Taxol. After treating the cells for varying duration, the culture medium was replaced with fresh serum-free medium containing 10% CCK-8 and cells were incubated for another 80 min at 37°C in the dark, and then the absorbance was measured at 490 nm. Results were reported as the ratio of cell proliferation/ inhibition, and the tests were repeated at least three times. The inhibition of proliferation rate (%) = (1- Ab (absorbance) value of the experimental group / Ab value of the control group) × 100%.

### Quantitative real-time polymerase chain reaction (qRT-PCR) assay

Total RNA was extracted from MCF-7 cells and MCF-7 /Taxol cells using Trizol (Sangon Biotech). The cDNA was synthesized using ReverTra Ace qPCR RT Master Mix (TOYOBO CO., LTD) according to the manufacturer’s instructions. Quantitative real-time PCR was performed on an ABI 7500 real-time system (Applied Biosystems, USA) using SYBR^®^ Green Realtime PCR Master Mix (TOYOBO CO., LTD.) and gene-specific primers according to the manufacturer’s protocol. Data were calculated using the 2^-ΔΔct^ method based on internal control. The relative expressions of ABCB1 and GSDME mRNA were normalized to GAPDH expression. The dataset was generated by at least three independent experiments. Primer sequences are listed in Table 1.

**Table 1:** Polymerase chain reaction (PCR) primers.

Name	Forward primer	Reverse primer
ABCB1	5’-GTGGGAAGAGCACAACA-3’	5’-GGTGGCAAACAATACAGG-3’
GSDME	5’-AGAAACCCTGTGCTCCA-3’	5’-TGACATTCCCATCCTCC-3’
GAPDH	5’-AGAACGGGAAGCTTGTCATC-3’	5’-CATCGCCCCACTTGATTTTG-3’

### Western blotting analyse

The harvested cells were washed twice with cold PBS (Solarbio), and then lysed with RIPA buffer containing phenylmethanesulfonyl fluoride (PMSF) for 30 min on ice. The lysates were centrifuged 12000 rpm for 10 min at 4° C, and the supernatants were collected. Protein concentration was determined using the BCA protein assay kit (Thermo Fisher Scientific, Rockford, USA). According to the targeted molecular weight, equal quantity of protein (20ug) samples were applied to 8% to 12% SDS-polyacrylamide gels and then transferred to PVDF membranes. Blocked with 5% non-fat milk in 1 × Tris-buffered saline containing 0.1% Tween-20 (TBST) for 1h at room temperature. Membranes were incubated overnight with specific primary antibodies against p-glycoprotein (p-GP) (1: 2000 dilution; Abcam, USA), GSDME (1: 10000 dilution, Abcam, USA), and GAPDH (1: 1000 dilution; Hangzhou Xianzhi, China). The membrane was then washed three times with TBST. After incubation with the corresponding secondary antibodies, their intensity was quantified using the Odyssey infrared imaging system (Li-Cor Inc., Lincoln, NE, USA) and Odyssey v3.0 software.

### Methylation profiling by MeDIP-Seq

To determine methylation levels, MeDIP was performed using 1ug of DNA isolated from breast cancer cells. MeDIP sequencing was performed by KangCheng bio-tech, Shanghai, China. DNA samples were fragmented to a range of 200-800bp by means of diagenetic biodiagenesis. Depending on the manufacturer’s instructions, approximately 1ug of DNA fragments were attached to Illumina’s genomic adapters and the ligated DNA was further immunoprecipitated by the anti-5-Methylcytosine antibody. The enriched DNA was amplified by PCR and purified by AMPure XP beads. The library was quantitatively evaluated using Agilent Bioanalyzer 2100 (Agilent Technologies, California, USA). Then the library was denatured by 0.1m NaOH to produce single-stranded DNA molecules, which were captured on Illumina flow cells and amplified in situ. Finally, the library was sequenced on Illumina NovaSeq 6000 according to the NovaSeq 6000 S4 reagent kit (300 cycles) protocols.

### Processing of MeDIP-Seq data

The raw sequencing data generated by Illumina NovaSeq 6000 was used for the following analysis. Trimmed reads (trimmed5^’^, 3^’^ -, adaptor base) were aligned to the reference genome. Referring to alignment statistical analysis (mapping ratio), we determined whether the sequencing results could be utilized for subsequent analysis. If possible, aligned reads were used for peak calling. LncRNA, mRNA and Small ncRNA associated MeDIP-enriched regions (peaks) with statistically significant were recognized to each sample, using a q-value threshold of 10^−5^ by MACS2. LncRNA, mRNA and Small ncRNA associated MeDIP-enriched regions (peaks) were annotated by the nearest gene using the latest UCSC RefSeq database. LncRNA, mRNA and Small ncRNA associated differentially Methylated regions (DMRs) within the promoter between two groups with statistically significant were identified by diffReps (Cut-off: log2FC≥1, p-value≤10^−4^). Both Enhancer and SuperEnhancer—related intergene DMRs were annotated by the nearest gene using the UCSC RefSeq database.

### MeDIP-quantitative PCR assay

Methylated DNA fragments were enriched by MeDIP technology and detected by real-time quantitative PCR. Finally, the methylation level of specific sites in breast cancer cells was detected by data analysis. The methylation status of candidate genes in experimental and control cells was quantitatively evaluated by combining real-time quantitative PCR with MeDIP-seq. The experiment was repeated three times. Primer sequences are listed in the Table 2.

**Table 2:** Polymerase chain reaction (PCR) primers.

Name	Forward primer	Reverse primer
GSDME	5’-TGTTTTCAGCCACCTAGTTTATG-3’	5’-GATCTCCTTTCCTGCAAGTCA-3’

### 5-Aza-2’-deoxycytidine (5-Aza-dC) treatment

MCF-7 / Taxol cells in the logarithmic growth phase were seeded onto 6-well cell culture plates at a density of 1×10^5^ cells per well in RPMI 1640 medium supplemented with 10% fetal bovine serum in a humidified atmosphere of 5%CO_2_ at 37℃for 24h. The cells were incubated in a medium containing an appropriate concentration of 5-Aza-dC (Sigma, Shanghai, China) for three days, and the medium was changed every day. These treated cells were used for follow-up experiments.

### LDH assay

A lactate dehydrogenase (LDH) cytotoxicity assay kit (Beyotime Biotech) was used to evaluate the integrity of the plasma membrane by the release of cytoplasmic enzyme LDH. Cells were subjected to different treatments, and then the cell culture medium was collected for examination according to the manufacturer’s instructions. LDH can catalyze lactic acid to produce pyruvate and NADH, which restores the water-soluble tetrazolium salt to formazan and shows up as orange in alkaline solutions. After the reaction, the absorbance was measured at a wavelength of 490 nm. According to the manufacturer’s instructions, the LDH release (%) was calculated by the following formula = (OD_Test_− OD_Control_) / (OD_Max_− OD_Control_) × 100%. (OD_Test_ indicates the absorbance value of the experimental group; OD_Control_ indicates the absorbance value of the control group; OD_Max_ indicates the absorbance value of the positive group).

### Annexin V-FITC staining

Cells were seeded overnight into 6-well plates at a density of 1×10^5^ cells per well and treated with Taxol for 24h. Cells were collected using Tryp E Express Enzyme, centrifuged at 1000 rpm for 5 min, and washed twice with cold PBS. The following staining procedures were performed with Annexin V-FITC and Propidium Iodide (PI) according to the manufacturer’s instructions. After resuspension with 100uL 1× Binding Buffer, cells were incubated with 5ul Annexin V-FITC and 5ul PI for 15min at room temperature in the dark. Resuspend cells with 400 μl 1× Binding Buffer. Finally, stained cells were analyzed by flow cytometry (BD Biosciences, San Jose, CA, USA).

### UALCAN dataset analysis

UALCAN enables researchers to perform in silico validation of potential genes of interest, providing gene expression and patient survival information. In this study, GSDME was included in the UALCAN data set to explore its impact on multidrug resistance in breast cancer.

### Statistical analysis

Statistical analysis was performed using SPSS 23.0 software. All results were obtained from at least three independent experiments and reported as mean ± SD. Comparisons between two groups were made with Student’s t-test, comparisons between multiple groups were performed using one-way analysis of variance (ANOVA), and pairwise comparisons were performed using LSD-t test. For all data, P <0.05 was considered statistically significant.

## Results

### ABCB1/ p-GP are upregulated in MCF-7 /Taxol cells

**Fig 1:**
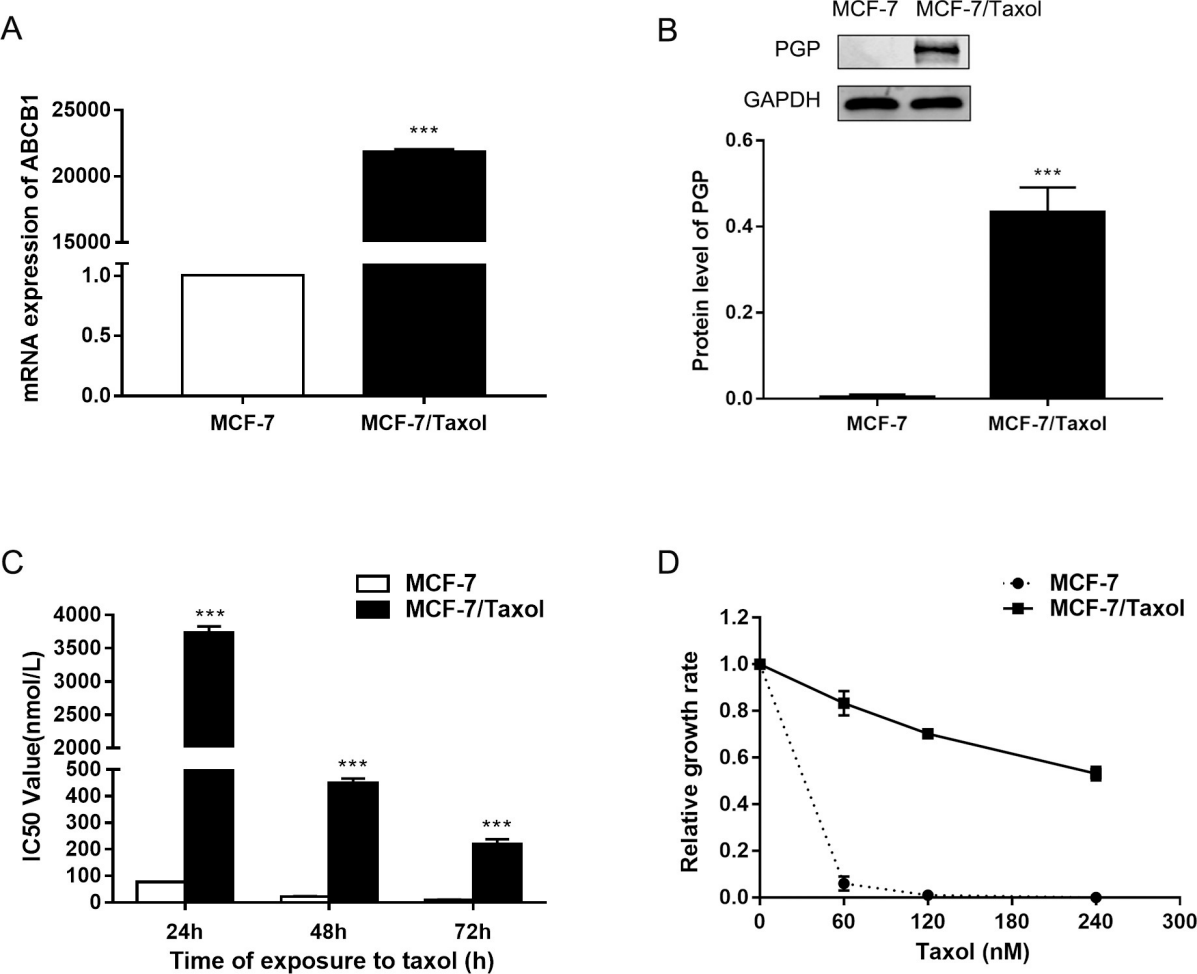
The expression levels of ABCB1 and p-GP are up-regulated in breast cancer MCF-7/Taxol cells. A. Expression levels of ABCB1 mRNA in MCF-7 and MCF-7/Taxol cells were determined by qRT-PCR. GAPDH was used as an internal control. B. Expression levels of p-GP in MCF-7 and MCF-7/Taxol cells were determined by Western blotting. C. Cell proliferation was determined by CCK-8 assay after treatment with Taxol at different time- points (24, 48 and 72h). D. MCF-7 and MCF-7/Taxol cells were cultured in regular medium with different Taxol concentrations (60, 120, 180, 240 and 300 nM), cell number was counted after 72 hours of Taxol treatment. Relative growth inhibition was normalized to cell number of MCF-7 cells without Taxol treatment. Data are expressed as the mean ± SD of three independent experiments. ***P<0.001 *vs* control group.

The ABCB1 gene was reported to be up-regulated in multidrug resistant (MDR) breast cancer cells [32, 33]. Therefore, we first tested the expression of ABCB1 gene in MCF-7 /Taxol cells and MCF-7 cells by qRT-PCR analysis. The results showed that the mRNA expression level of ABCB1 gene increased in MCF-7 /Taxol cells (Fig 1A). To further confirm these findings, we compared the protein levels of p-GP in these two cells using western blot analysis. We found that p-GP protein levels in MCF-7 /Taxol cells were up-regulated in line with changes in their mRNA levels (Fig 1B). We also noted that MDR gene expression was almost undetectable in MCF-7 cells. These results suggested that the expression of p-GP may be linked to Taxol resistance in breast cancer cells. Next, we examined the IC50 of MCF-7 /Taxol cells and MCF-7 cells treated with Taxol at different concentrations (Fig 1C). MCF-7 cells can completely induce cell death after 72h of Taxol treatment, while MCF-7 /Taxol cells can survive even after exposure to 240nM Taxol (Fig 1D). It confirmed that MCF-7 /Taxol cells were resistant to Taxol. The experimental results showed that the Resistance Index (RI) of MCF-7/Taxol cells was greater than 15, which had been successfully induced into a high-resistance model. (Resistance Index (RI) = IC50_(MCF-7/Taxol)_ /IC50_(MCF-7)_).

### GSDME expression is regulated by epigenetic mechanisms in breast cancer cells

**Fig 2:**
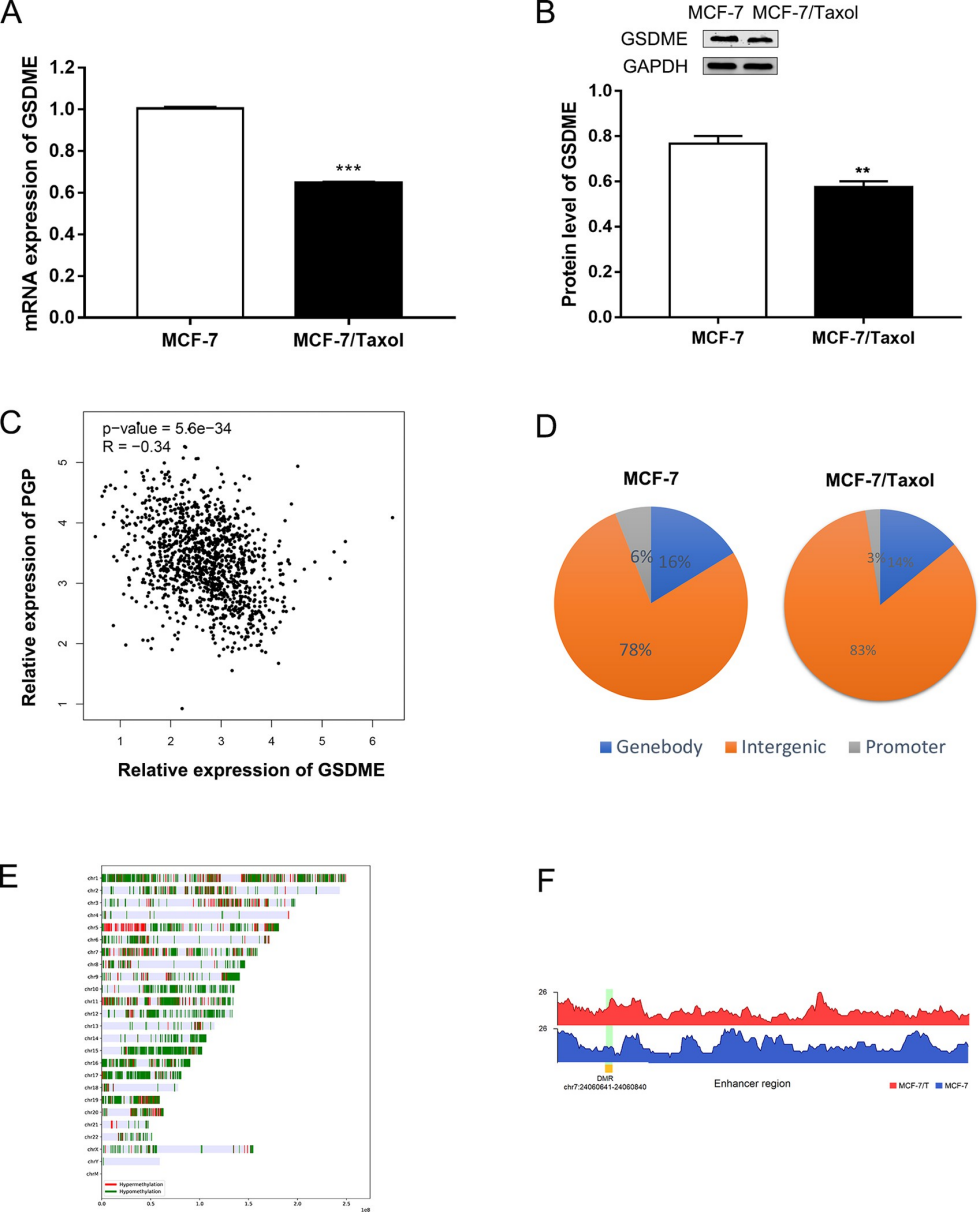
GSDME expression is regulated by epigenetic mechanisms in breast cancer cells. A and B. qRT-PCR and Western blot analysis showed GSDME expression in MCF-7 and MCF-7/Taxol cells. **P<0.01, ***P<0.001 *vs* control group. C. Pearson’s correlation analysis of the relative expression levels of GSDME and the relative p-GP protein expression levels in the same set of tissues. D. The distribution map of methylation peaks in the whole genome. E. The distribution of DMRs in chromosomes. F. Differential methylation peaks in the enhancer region.

First, we detected the expression of GSDME using qRT-PCR and WB analyses in MCF-7 / Taxol cells and MCF-7 cells. The results showed that compared with MCF-7 cells, the expression of GSDME was down-regulated in MCF-7 / Taxol cells at the level of transcription and protein (Fig 2A and 2B). In addition, this study assessed the correlation between GSDME and p-GP using the UALCAN dataset. Correlation analysis showed that tissues with higher GSDME expression had lower p-GP protein (Fig 2C) (R = −0.34, P <0.01), which was consistent with the results of previous research [31].

To detect whether the low expression of GSDME in MCF-7 / Taxol cells is regulated by epigenetic mechanisms, we performed Methylated DNA immunoprecipitation-sequencing (MeDIP-seq) on MCF-7 / Taxol cells and MCF-7 cells. We plotted all the methylation signals in these cells. According to the location of methylation peaks in the genome, the methylation peaks were divided into three categories: promoter peaks, genebody peaks and intergenic peaks. We found that most of the methylation peaks were located in the intergenic region, and only a few methylation peaks were located in the promoter region (Fig 2D). MCF-7/Taxol cells had lower methylation levels compared to MCF-7 cells (Table 3). These results suggest that DNA methylation patterns differ between the two groups of cells. Next, we plotted the distribution of DMRs on all chromosomes. As shown in (Fig 2E), hypermethylated and hypomethylated DMRs signals were mapped to the entire genome. Interestingly, we found differential methylation peaks of GSDME in the enhancer region (Fig 2F). It can be seen that the degree of methylation of GSDME in the enhancer region of MCF-7 / Taxol cells was higher than that of MCF-7 cells, which is consistent with the expression of GSDME in these cells. These results indicated that GSDME expression is regulated by epigenetic mechanisms in breast cancer cells.

**Table 3:** The number of methylation peaks in the genome.

Gene region	Peak numbers	
	MCF-7/Taxol	MCF-7
Genebody	845.6666667	1537.333333
Intergenic	5081	7351.666667
Promoter	150.3333334	566.3333334

### Decitabine can demethylate breast cancer cells and increase GSDME expression

**Fig 3:**
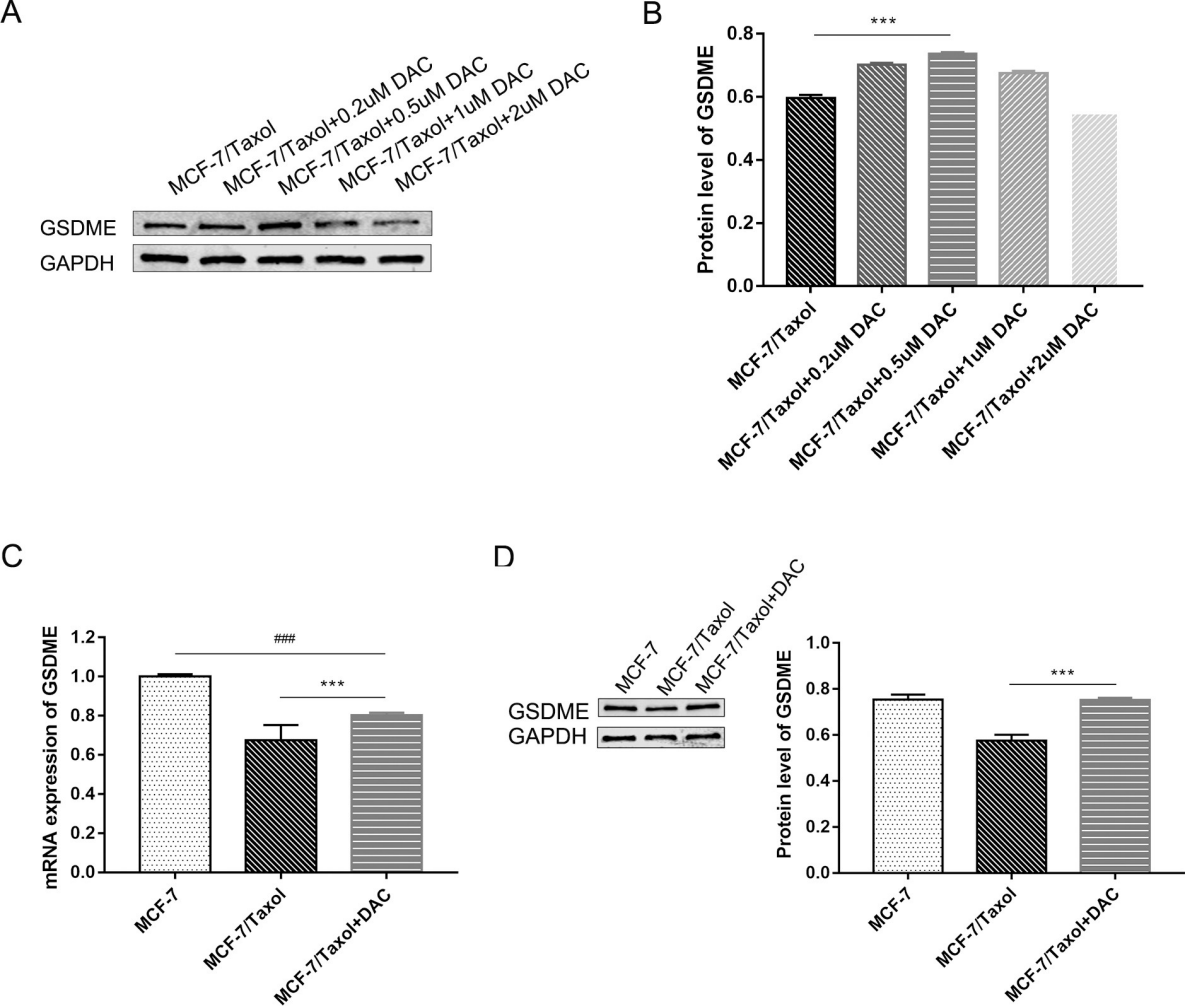
GSDME expression in breast cancer after decitabine treatment. A and B. Breast cancer cell lines were treated with 0.2 uM, 0.5 uM, 1 uM and 2 uM decitabine for 72h and GSDME expression levels were quantified by Western blot analysis. The results showed that 0.5 uM decitabine treatment significantly increased the expression of GSDME in MCF-7 /Taxol cells. C and D. GSDME expression after 0.5 uM decitabine was determined by qRT-PCR and Western blot analysis. Data are expressed as the mean ± SD of three independent experiments. ***P<0.001 *vs* control group, ^###^*P*<0.001 *vs* control group.

The GSDME methylation level in the enhancer region of breast cancer cells has been identified. To further explore the role of GSDME in chemotherapy resistance, MCF-7 /Taxol cells were treated with the demethylation agent to express GSDME protein. Cells were treated with 0.2uM, 0.5uM, 1.0uM and 2.0uM decitabine for 72h, and then the expression of GSDME was detected by PCR and WB assays. It can be seen from the experimental results that 0.5 uM decitabine treatment significantly increased the expression of GSDME in MCF-7 /Taxol cells (Fig 3A and 3B). Therefore, this concentration was selected to treat cells to construct the demethylation model of MCF-7/Taxol cells.

Demethylation of cells increased the expression of GSDME in breast cancer-resistant cells. To determine the extent to which GSDME expression increased before and after the treatment of decitabine treatment, we performed qPCR and WB analyses on three groups of breast cancer cells. The results showed that the expression of GSDME in the decitabine treated group was higher than that in the untreated group but still lower than that in the MCF-7 cells group (Fig 3C and 3D). Therefore, DNA methylation is not the only factor that reduces GSDME expression, and further studies may be needed to improve GSDME expression in MCF-7/Taxol. But what we do know is that decitabine can demethylate breast cancer MCF-7/Taxol cells and increase GSDME expression.

### Effects of combination decitabine and Taxol reduce the proliferation of MCF-7/Taxol cells and increase their susceptibility to chemotherapy

Through CCK-8 assay, it can be recognized that the IC50 value of MCF-7 /Taxol cells to Taxol was much higher than that of MCF-7 cells. In other words, the rate of inhibition of MCF-7/Taxol was lower under the same treatment conditions. It was found that increasing the expression of GSDME induced tumor cell pyroptosis. Therefore, we treated MCF-7/Taxol cells with demethylated drugs and detected cell proliferation by CCK-8 assay to see if cell growth was affected.

**Fig 4:**
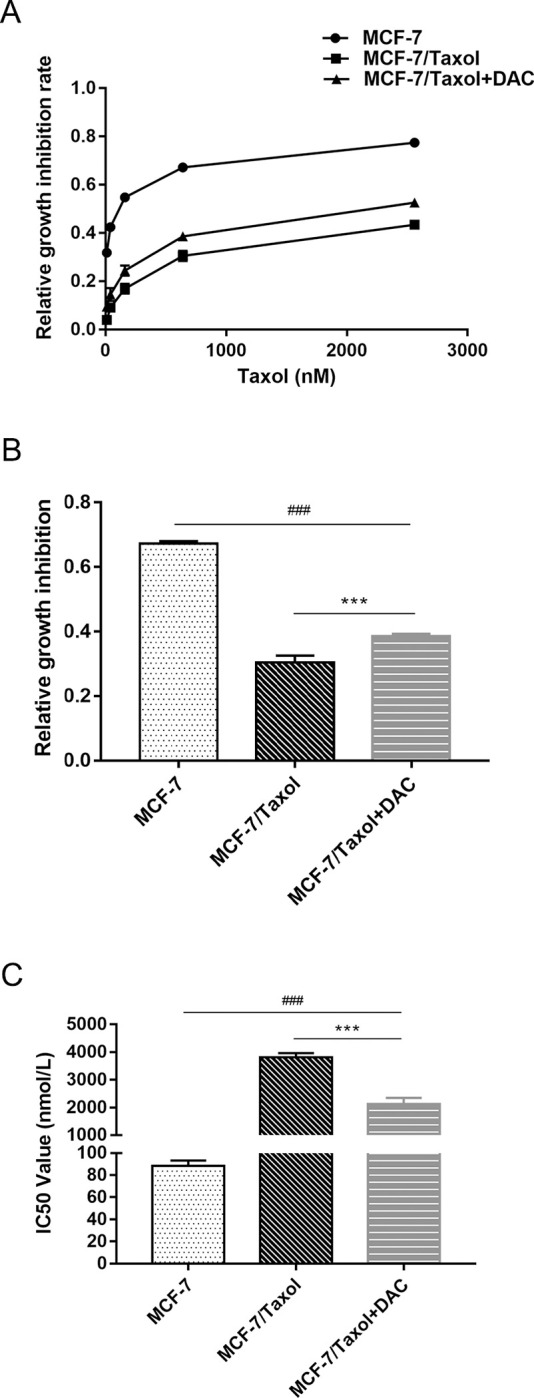
Combined decitabine and Taxol decreases MCF-7/Taxol cells proliferation and can improve their chemosensitivity. A. Cell growth inhibition rate of breast cancer cell lines was determined by CCK-8 assay after demethylation with 0.5uM decitabine and treatment with different concentrations of Taxol for 24h. B. Cell growth inhibition rate was showed by CCK-8 assay after demethylation with 0.5 uM decitabine and treatment with 640 nM Taxol. C. Cell proliferation was determined after treatment with different concentrations of Taxol for 72h. Data are expressed as the mean ± SD of three independent experiments. ***P<0.001 *vs* control group, ^###^*P*<0.001 *vs* control group.

Treatment of MCF-7/Taxol cells with decitabine for 72h as a drug treatment group. A series of concentration gradients of Taxol was applied to MCF-7 cells, MCF-7 /Taxol cells, and drug-treated cells for 24h. We found that the higher the drug concentration, the higher the inhibition rate of cells in each group. The inhibition rate of cells in each group was most significantly different at the concentration of 640nM Taxol (Fig 4A). Therefore, we chose the concentration of 640nM Taxol as the treatment concentration in subsequent experiments. MCF-7 cells, MCF-7 /Taxol cells and decitabine treated cells were treated with this concentration, respectively. After 24h of drug treatment, the proliferation capacity of cells was observed by CCK-8 assay to reflect the sensitivity of cells to drugs (Fig 4B). Then, we evaluated the effect of GSDME expression on the proliferation of MCF-7 /Taxol cells. The results showed that under the same treatment conditions, IC50 of decitabine treated group cells was significantly increased compared with MCF-7 /Taxol cells. However, compared with MCF-7 cells, the IC50 of the decitabine treated cells was slightly lower (Fig 4C). In other words, MCF-7 /Taxol cells had the highest proliferation capacity, while MCF-7 cells had the lowest. The proliferation capacity of cells is inversely proportional to the sensitivity of cells to chemotherapy drugs. As demonstrated by previous experimental results, cells treated with decitabine are more sensitive to chemotherapeutic drugs due to increased expression of GSDME.

### Combined treatment with decitabine and Taxol can induce pyroptosis in MCF-7/Taxol cells

**Fig 5:**
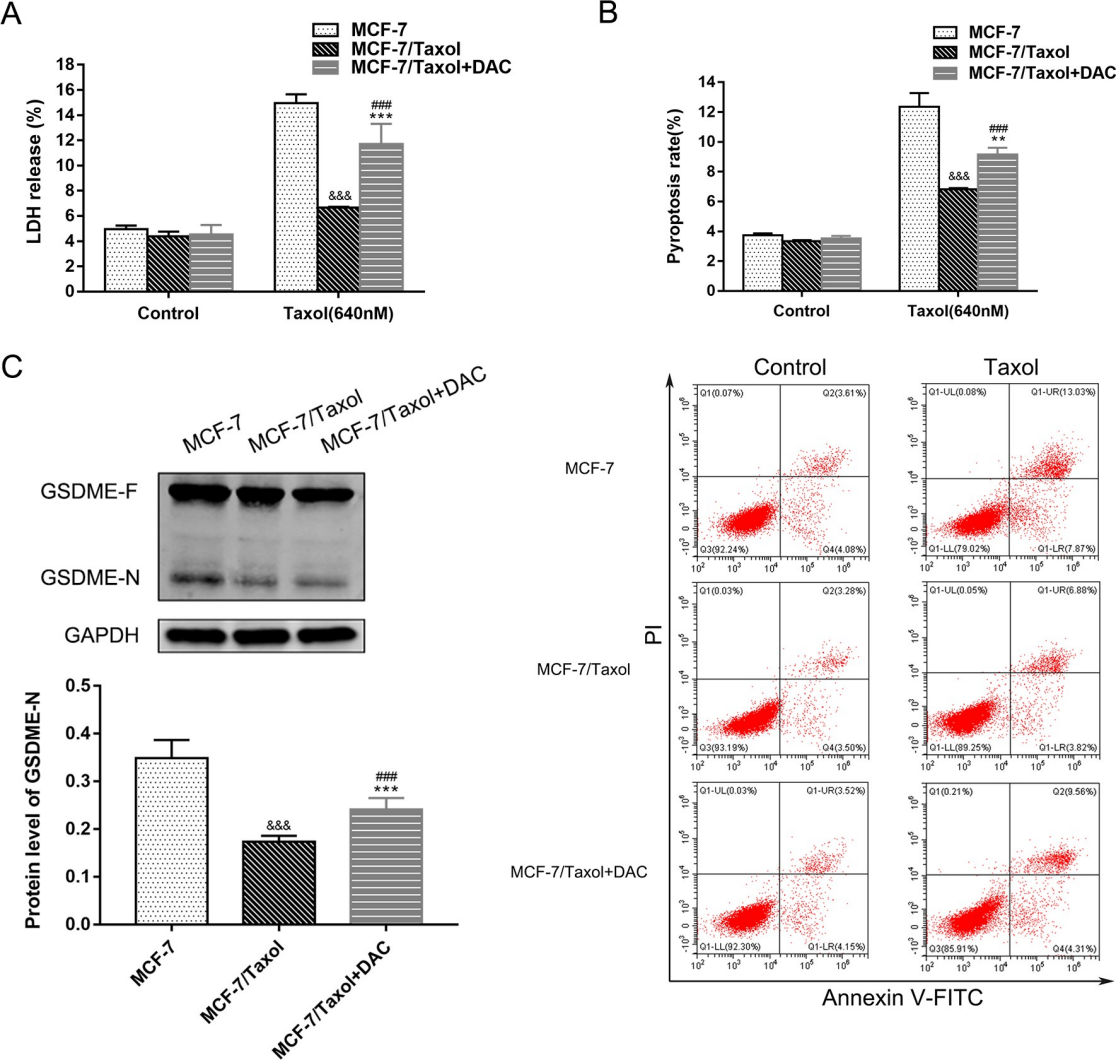
Combined decitabine and Taxol induce MCF-7/Taxol cells pyroptosis. A. The integrity of cell membrane was determined by the LDH release assay of breast cancer cells with 640nM Taxol. B. Flow cytometry was used to detect the incidence of pyroptosis in breast cancer cells treated with 640nM Taxol for 24h. C. The expression of GSDME-N, an active fragment of the key protein of pyroptosis, was detected by Western blot analysis. Data are expressed as the mean ± SD of three independent experiments. **P<0.01 *vs* control group, ***P<0.001 *vs* control group, ^###^*P*<0.001 *vs* control group, ^&&&^*P*<0.001 *vs* control group.

We found that increased GSDME expression can improve the sensitivity of cells to chemotherapy drugs, but it is not clear whether GSDME expression level is directly related to chemotherapy sensitivity. Resistance to cancer drugs is well known to be associated with low sensitivity to apoptosis [34]. However, recent studies have shown that chemotherapeutic drugs can induce pyroptosis in tumor cells with high GSDME expression [2, 28]. Therefore, we treated MCF-7 cells, MCF-7 /Taxol cells and decitabine treated cells with 640nM Taxol for 24h, and then detected the occurrence of pyroptosis by the LDH release assay and flow cytometry. These results showed that the LDH release level and pyroptosis rate of MCF-7 cells were higher than those of the other two groups (Fig 5A and 5B). Meanwhile, the LDH release level and pyroptosis rate of cells treated with decitabine were higher than those of the untreated group. We can say that Taxol can induce pyroptosis in MCF-7 cells with high expression of GSDME protein, so MCF-7 cells are highly sensitive to Taxol. To verify that GSDME is indeed involved in the occurrence of pyroptosis, we also examined the activation of GSDME. According to the results of WB analyses, the expression of GSDME-N was proportional to the occurrence of pyroptosis (Fig 5C, **S2 and S3 Figs**), and GSDME did influence the sensitivity of cells to chemotherapy drugs by inducing pyroptosis. These results suggest that the treatment of MCF-7 /Taxol cells with low expression of GSDME with the demethylated drug decitabine improves the chemical sensitivity of it to Taxol by reversing the expression of GSDME to induce pyroptosis.

## Discussion

In the past few decades, remarkable advances have been made in the prevention, detection, and treatment of cancer. Taxol, an important chemical in the treatment of cancer, including breast cancer, has been able to improve survival rates [35]. But there are problems with chemotherapy, such as secondary drug resistance [9, 36]. The risk of tumors acquiring chemotherapy resistance (multidrug resistance) is a major barrier to successful treatment of various cancers, including breast cancer. Therefore, the emergence of chemotherapy resistance makes some patients less sensitive to Taxol, which leads to poor prognosis. The mechanism of drug-resistance is complex, among which the abnormal expression of Multidrug Resistance Gene (MDR) is an important reason for the drug-resistance of cells. The MDR1 gene encodes ATP-dependent membrane transporter (p-glycoprotein, p-GP), which can directly bind to chemotherapeutic drugs, promote the pumping of drugs from cells and reduce the cytotoxicity of drugs. At present, in order to solve the phenomenon of chemotherapy resistance in breast cancer, the low concentration gradient induction method is mainly used to construct experimental models of drug-resistant strains for in vitro research.

One of the characteristics of tumors is escaping from apoptosis. So induced pyroptosis is particularly important in the treatment of anti-apoptotic tumors [37]. In this study, we found that GSDME expression promoted Taxol-induced cell death. Importantly, GSDME increased the sensitivity of MCF-7 cells to Taxol by inducing pyroptosis. However, the presence of DNA methylation in MCF-7 /Taxol cells reduced GSDME expression, which led to apoptosis rather than pyroptosis after Taxol treatment. In other words, MCF-7 /Taxol cells were less sensitive to Taxol. Therefore, we conclude that GSDME methylation inhibits pyroptosis and thus reduces the sensitivity of MCF-7 /Taxol cells to Taxol. This finding suggests that tumor cells may down-regulate GSDME expression to prevent cell death, providing new insights into chemotherapeutic resistance.

Studies have found that the expression level of GSDME is one of the key factors affecting the chemosensitivity of breast cancer cells [38]. Drug-resistance transformation in tumor cells is usually associated with increased drug dose or multidrug combination administration, which often makes cells multidrug resistant. Through the results of this study, we found that the expression of p-GP was negatively correlated with GSDME. This finding is very attractive, suggesting that we can study how the p-GP gene affects the expression of GSDME to produce drug-resistant phenomenon. What are the mechanisms that alter biological function in breast cancer drug-resistant cells? What other epigenetic changes exist in drug-resistant cells? These problems need to be explored and discovered in the future.

The discovery of pyroptosis has aroused widespread concern in the academic community, but subsequent studies have focused on the interaction with other proteins, and little is known about the clinical significance of the key molecule of pyroptosis, GSDME. Recent studies have suggested that GSDME may play several important roles in the early diagnosis of breast cancer [2, 39]. Our data also indicates that GSDME is cleaved by stimulation with Taxol, which lead to pyroptosis. This discovery has changed our understanding of programmed cell death. Moreover, the expression level of GSDME determines the form of cell death in caspase-3 activated cells stimulated by chemotherapeutic drugs, thereby influencing the sensitivity of cells to chemotherapeutic drugs.

We used a highly sensitive method enriched involving enrichment by MeDIP to detect methylated DNA fragments. High-throughput sequencing enabled detect abnormal methylation sites in the genomes of breast cancer cells [40]. Our data set covers almost the entire genome and is deep enough to identify differential methylation regions, thus providing high resolution and reproducibility and proving that MeDIP-seq is an economical approach for comparative analysis of DNA methylation in mammals [41]. Therefore, this study provides important new insights into the biological significance of DNA methylation.

Most studies of cancer methylation are limited to functionally important promoters, and they ignore other areas [42]. However, extensive hypermethylation of many non-promoter regions was found in our analysis. Hypermethylation occurs not only at the proximal promoter but also at exons and introns, including regions at the distal end of TSS, where changes in gene methylation may affect expression [43]. In this study, we found GSDME genes with differential methylation in the enhancer region. That is, there was a higher level of GSDME methylation in the enhancer region in MCF-7 /Taxol cells. At the same time, we verified the relationship between methylation and expression of enhancer genes by PCR and WB analyses. It is concluded that GSDME expression is decreased in MCF-7 /Taxol cells due to high methylation in the enhancer region.

Inflammation bodies are produced during the process of pyroptosis, which can induce pyroptosis of tumor cells and inhibit its proliferation. However, the accumulation of inflammasomes will form a microenvironment for the growth of tumor cells, which are conducive to the proliferation of tumor cells [14]. Therefore, pyroptosis has a dual mechanism of promoting and inhibiting tumorigenesis. Further exploration of the mechanism of pyroptosis in different tumor cells can provide new ideas for the treatment. However, little is known about the putative role of pyroptosis in the development of drug resistance in cancer cells. In this study, we found that Taxol plays an anticancer role in inducing pyroptosis by cleaving GSDME protein, suggesting that pyroptosis may be related to the sensitivity of Taxol. However, some tumor cells express little GSDME. It has been suggested to reverse the GSDME silence by using decitabine [15, 44]. Therefore, we used low decitabine to increase the expression of GSDME in MCF-7/Taxol cells. Decitabine is known to degrade the DNMT1 protein when administered at high concentrations and cause DNA damage in the context of high doses and short-term treatment [45, 46]. High concentrations of decitabine can decrease GSDME expression in cells, because decitabine is cytotoxic and can kill cells if the concentration is too high. Therefore, we chose 0.5uM decitabine for subsequent experiments. At this concentration, the expression of GSDME was the highest in drug-resistant cells, which also ruled out the damage of high concentrations of drugs to cells. A recent clinical trial in patients with advanced lung cancer also used a low-dose of decitabine and produced stable responses [47]. Future studies need to test doses of decitabine or decitabine-like drugs in clinical trials in breast cancer patients.

Upon stimulation of Taxol, caspase-3 activated GSDME. Ultimately, activated GSDME induced pyroptosis. The results showed that increasing the expression of GSDME improved the sensitivity of MCF-7/Taxol cells to Taxol by inducing pyroptosis. Further evidence suggests that GSDME is indeed involved in tumor biology, and its level of expression may lead to changes in treatment strategies [18]. It is reasonable to speculate that pyroptosis is of great significance in the treatment of breast cancer with Taxol, and tumor cells prone to pyroptosis are more sensitive to chemotherapy drugs. The combination of demethylated drugs and chemotherapy drugs will be the focus of future research. Moreover, studies have shown that decitabine is more effective in combination with chemotherapeutic agents in the treatment of myelodysplastic syndrome and acute myeloid leukemia [48]. Decitabine /GSDME/ pyroptosis may be developed as a new method to overcome Taxol resistance in breast cancer. This discovery may provide a direction for the development of personalized and precise medicine for breast cancer in the future.

In summary, our study provides evidence that increased GSDME expression through DNA demethylation induces pyroptosis, thereby increasing the chemical sensitivity of MCF-7 / Taxol cells to Taxol. In other words, the combined treatment of decitabine and Taxol resulted in the upregulation of GSDME expression in breast cancer resistant cells, which induced pyroptosis and made the cells more sensitive to chemotherapy drugs. Our findings may help to better understand the molecular mechanisms of resistance in breast cancer and suggest that GSDME overexpression is a promising strategy to combat multidrug resistance. Future research in this area may suggest new strategies to better use GSDME-mediated pyroptosis to treat cancer.

## Supporting information

S1 Fig Pyroptosis: A type of programmed cell death mediated by the Gasdermin family. Pyroptosis is divided into two major pathways: one is Gasdermin-D (GSDMD) mediating pyroptosis after inflammatory caspases cleavage, and the other is GSDME converting caspase-3-induced apoptosis into pyroptosis. The former can be divided into Caspase-1-dependent classical pyroptosis pathway and Caspase-4/5/11-dependent non-classical pathway according to different activated Caspase. Pathogen-associated molecular patterns or risk-associated molecular patterns activate inflammasome sensor, which triggers the recruitment of Caspase-1 to form inflammasome. Caspase-1 directly cleaves GSDMD and cytokine precursors pro-IL-1β and pro-IL-18, producing the GSDMD-N terminal domain and promoting IL-1β and IL-18 maturation. The GSDMD-N domain targets cell membrane and aggregates to form membrane pores, thereby inducing pyroptosis. In the nonclassical pathway dependent on Caspase-4/5/11, Caspase-4/5 in human or Caspase-11 in mouse directly recognizes the cytoplasmic LPS of Gram-negative bacteria, leading to these inflammatory Caspase directly cleave GSDMD and inducing pyroptosis. In this study, we found that GSDME converts caspase-3-induced apoptosis to pyroptosis. Ligand binding to the receptor triggers assembly of FADD and pro-Caspase-8 complex, which results in Caspase-8 activation. Cytochrome C is released through mitochondrial activation, causing Caspase-9 activation. Activated Caspase-8 and Caspase-9 cleave downstream caspase-3 and mediate apoptosis. However, in GSDME positive cells, chemotherapeutic drugs activate apoptosis-related caspase-3, and the activated caspase-3 lyses GSDME to generate the GSDME-N domain, which targets plasma membrane and induces pyroptosis.
(TIF)

S2 Fig The expression of GSDME-N, an active fragment of the key protein of pyroptosis, was detected by Western blot analysis. Blot 1.
(TIF)

S3 Fig The expression of GSDME-N, an active fragment of the key protein of pyroptosis, was detected by Western blot analysis. Blot 2.
(TIF)
