# Supplementary figures and images for: Promoting GSDME expression through DNA demethylation to increase chemosensitivity of breast cancer MCF-7 / Taxol cells
Source: PLoS One. 2023 Mar 3;18(3):e0282244. doi: 10.1371/journal.pone.0282244 (PMC9983855; doi:10.1371/journal.pone.0282244)

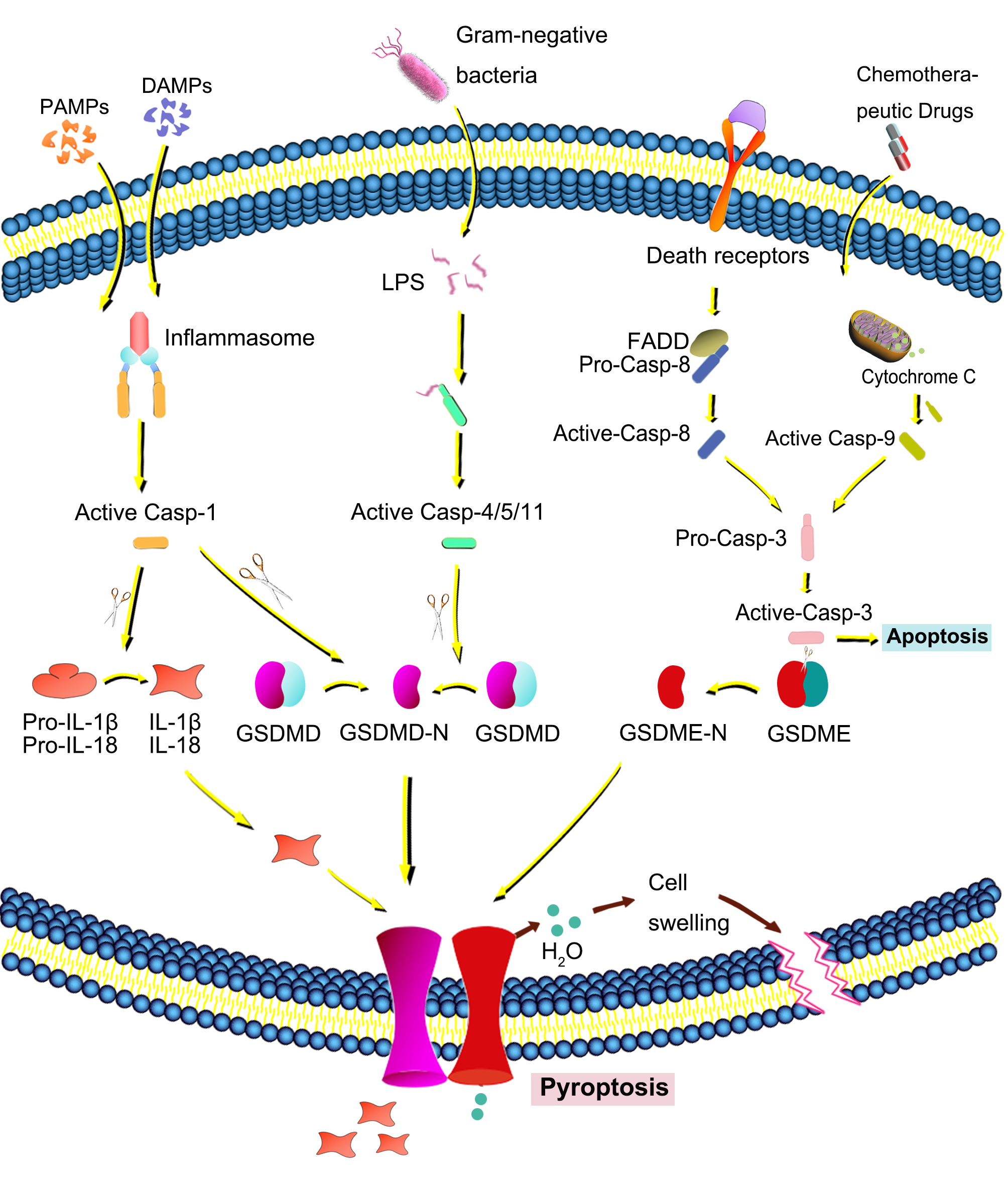

Supplement: S1 Fig — Pyroptosis is divided into two major pathways: one is Gasdermin-D (GSDMD) mediating pyroptosis after inflammatory caspases cleavage, and the other is GSDME converting caspase-3-induced apoptosis into pyroptosis. The former can be divided into Caspase-1-dependent classical pyroptosis pathway and Caspase-4/5/11-dependent non-classical pathway according to different activated Caspase. Pathogen-associated molecular patterns or risk-associated molecular patterns activate inflammasome sensor, which triggers the recruitment of Caspase-1 to form inflammasome. Caspase-1 directly cleaves GSDMD and cytokine precursors pro-IL-1β and pro-IL-18, producing the GSDMD-N terminal domain and promoting IL-1β and IL-18 maturation. The GSDMD-N domain targets cell membrane and aggregates to form membrane pores, thereby inducing pyroptosis. In the nonclassical pathway dependent on Caspase-4/5/11, Caspase-4/5 in human or Caspase-11 in mouse directly recognizes the cytoplasmic LPS of Gram-negative bacteria, leading to these inflammatory Caspase directly cleave GSDMD and inducing pyroptosis. In this study, we found that GSDME converts caspase-3-induced apoptosis to pyroptosis. Ligand binding to the receptor triggers assembly of FADD and pro-Caspase-8 complex, which results in Caspase-8 activation. Cytochrome C is released through mitochondrial activation, causing Caspase-9 activation. Activated Caspase-8 and Caspase-9 cleave downstream caspase-3 and mediate apoptosis. However, in GSDME positive cells, chemotherapeutic drugs activate apoptosis-related caspase-3, and the activated caspase-3 lyses GSDME to generate the GSDME-N domain, which targets plasma membrane and induces pyroptosis. (TIF) [file pone.0282244.s001.tif]

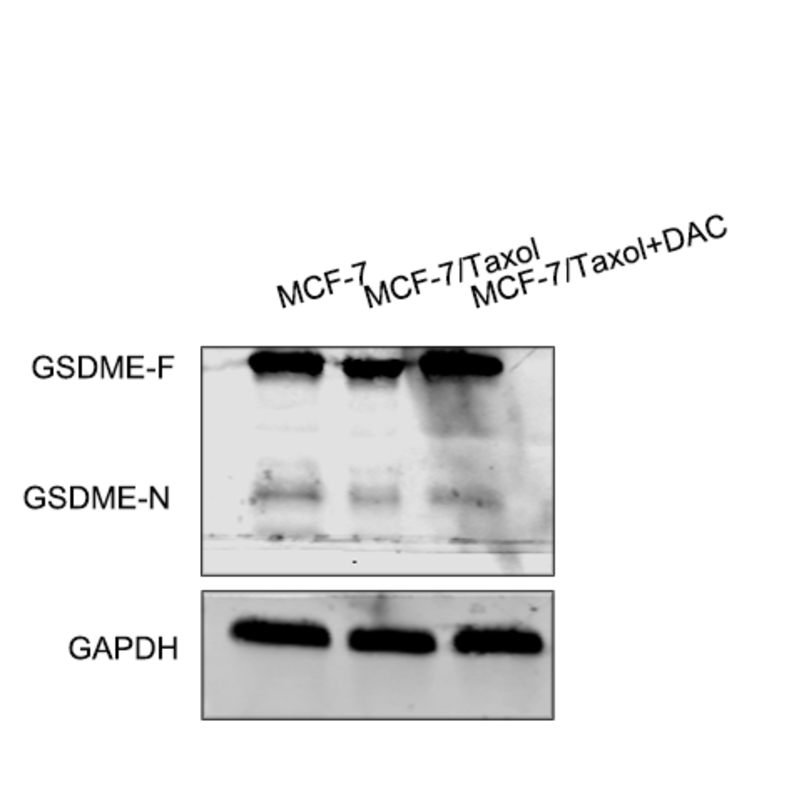

Supplement: S2 Fig — Blot 1. (TIF) [file pone.0282244.s002.tif]

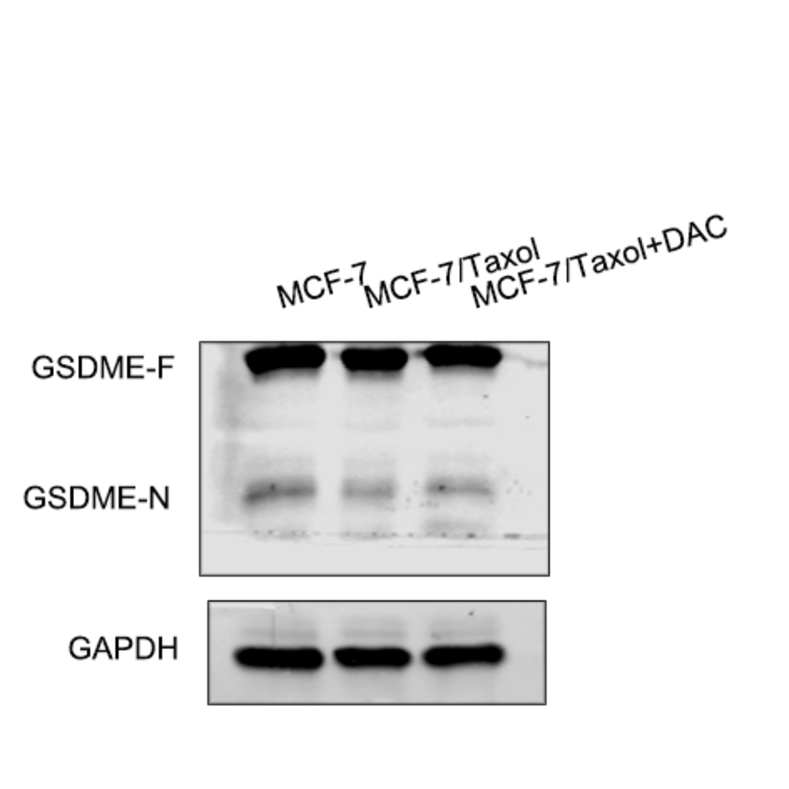

Supplement: S3 Fig — Blot 2. (TIF) [file pone.0282244.s003.tif]
